# The application of three-dimensional printed patient-specific drilling templates for expansive open-door laminoplasty: A single-center, prospective randomized controlled study

**DOI:** 10.3389/fsurg.2022.1084804

**Published:** 2023-01-09

**Authors:** Kangkang Huang, Xuelin Pan, Yuting Wen, Beiyu Wang, Chen Ding, Tingkui Wu, Xin Rong, Hao Liu

**Affiliations:** ^1^ Department of Orthopedics, West China Hospital, Sichuan University, Chengdu, China; ^2^ Department of Radiology, West China Hospital, Sichuan University, Chengdu, China

**Keywords:** laminoplasty, three-dimensional printing, template, hinge, cervical spondylosis

## Abstract

**Background:**

The choice of trough position in Expansive open-door laminoplasty (EOLP) mostly relied on bony landmarks and surgeons’ experience. The present study was to validate the efficacy of the three-dimensional (3D) printed patient-specific drilling templates with the function of locating and depth control for EOLP.

**Materials and Methods:**

A single-center, prospective randomized controlled study was conducted on the patients who underwent unilateral EOLP from August 2019 to December 2020. The 3D printed patient-specific drilling template was fabricated and used in the template group. All the EOLP were performed by a senior surgeon and a junior surgeon. The clinical outcomes and radiographic results were evaluated.

**Results:**

A total of 37 patients who completed the 12-month follow-up were analyzed. The clinical outcomes were significantly improved after surgery in both groups (*P* < 0.05). The visual analogue scale (VAS) scores were significantly lower in the template group at 12 months postoperatively (*P* < 0.05). The anteroposterior diameter, Pavlov's ratio and Open angle were all higher in the template group than those in the control group at 3 days and 12 months postoperatively (*P* < 0.05). The satisfaction of the trough position on both sides and incomplete fracture rate on the hinge side were higher in the template group based on the CT scans taken 3 days after surgery (*P* < 0.05). To the junior surgeon, the satisfaction and the incomplete fracture rate were significantly higher in the template group compared with those in the control group (*P* < 0.05).

**Conclusion:**

The application of 3D printed patient-specific drilling templates with the function of locating and depth control for EOLP could improve the outcome of neck pain relief and expand the decompression. It can also improve the satisfaction of the trough position on the open-door side and the hinge side and decrease the complete fracture rate on the hinge side, especially for the junior surgeon.

## Introduction

Expansive open-door laminoplasty (EOLP) has been widely performed for patients with cervical spondylotic myelopathy caused by multilevel disc herniation (MDH), congenital cervical stenosis (CCS) or ossification of the posterior longitudinal ligament (OPLL). Since Hirabayashi et al. (1) first described the EOLP in 1983, many authors have demonstrated that this technique is effective for neurological improvement in patients with spondylotic myelopathy (2, 3). The trough preparation is one of the most technic demanding procedures of the EOLP, requiring ideal trough position and precise bone removal. Nowadays, the choice of the trough position mostly relied on the bony landmarks and the surgeons' experience. However, complications such as complete fracture of the hinge side and insufficient decompression due to unsatisfactory trough position or excessive bone removal were reported range from 5.2%–57.6% in the literatures (4–6). Our previous study reported the safety and efficacy of the three-dimensional (3D) printed templates with the function of trough locating in the unilateral EOLP (7–9). We found that the templates could decrease the incidence of complete fracture on the hinge side through accurate locating. However, the depth of the cutting path still depended on the surgeon's experience. We subsequently modified the templates by adding the function of depth control. The trough position and cutting depth for both the hinge side and the open-door side were designed in the computer software based on the patients' computed tomography (CT) scans. The purpose of this study was to prospectively and randomly validate the efficacy of the modified 3D printed templates. The material of the template was acrylate resin which had been approved safety in patients by the United States Food and Drug Administration.

## Materials and methods

This was a single-center, prospective randomized controlled study started from August 2019. The study protocol was approved by the Ethics Committee of our Hospital. The inclusion criteria were: (1) the age was older than 18 years old; (2) cervical spondylosis due to MDH, CCS or OPLL refractory to conservative treatments for at least 6 weeks; (3) the diagnosis was confirmed by imaging (CT, magnetic resonance imaging, or radiography); (4) the K line was (+); (5) the surgical plan was the unilateral EOLP from C3 to C7 with Centerpiece mini-plate (Centerpiece TM Plate Fixation System, Medtronic Sofamor Danek, USA); and (6) the patient agreed to participate in this study and gave the written informed consent. The exclusion criteria were: (1) cervical trauma, deformity, primary tumor, cervical metastasis or infection; (2) prior cervical spine surgery; (3) mental illness. The patient was rejected if the surgical plan was changed. The target sample size was 20 patients in the template group and 20 in the control group. The timepoints for evaluation were pre-operation, 3 days postoperatively and 12 months postoperatively.

The patients were randomly assigned to the template group and control group through a random number generator. Patients with odd numbers were attributed to the template group and even numbers were assigned to the control group. In the template group, the EOLP was performed with the aid of the 3D printed patient-specific drilling templates. The treatment was blinded to the radiologists who were responsible for the image taking and parameters measurement.

All the EOLP were performed by two surgeons. One was a senior surgeon with a surgical experience of more than 20 years. The other one was a junior surgeon with a surgical experience of fewer than 5 years.

### EOLP template fabrication

**Figure 1:**
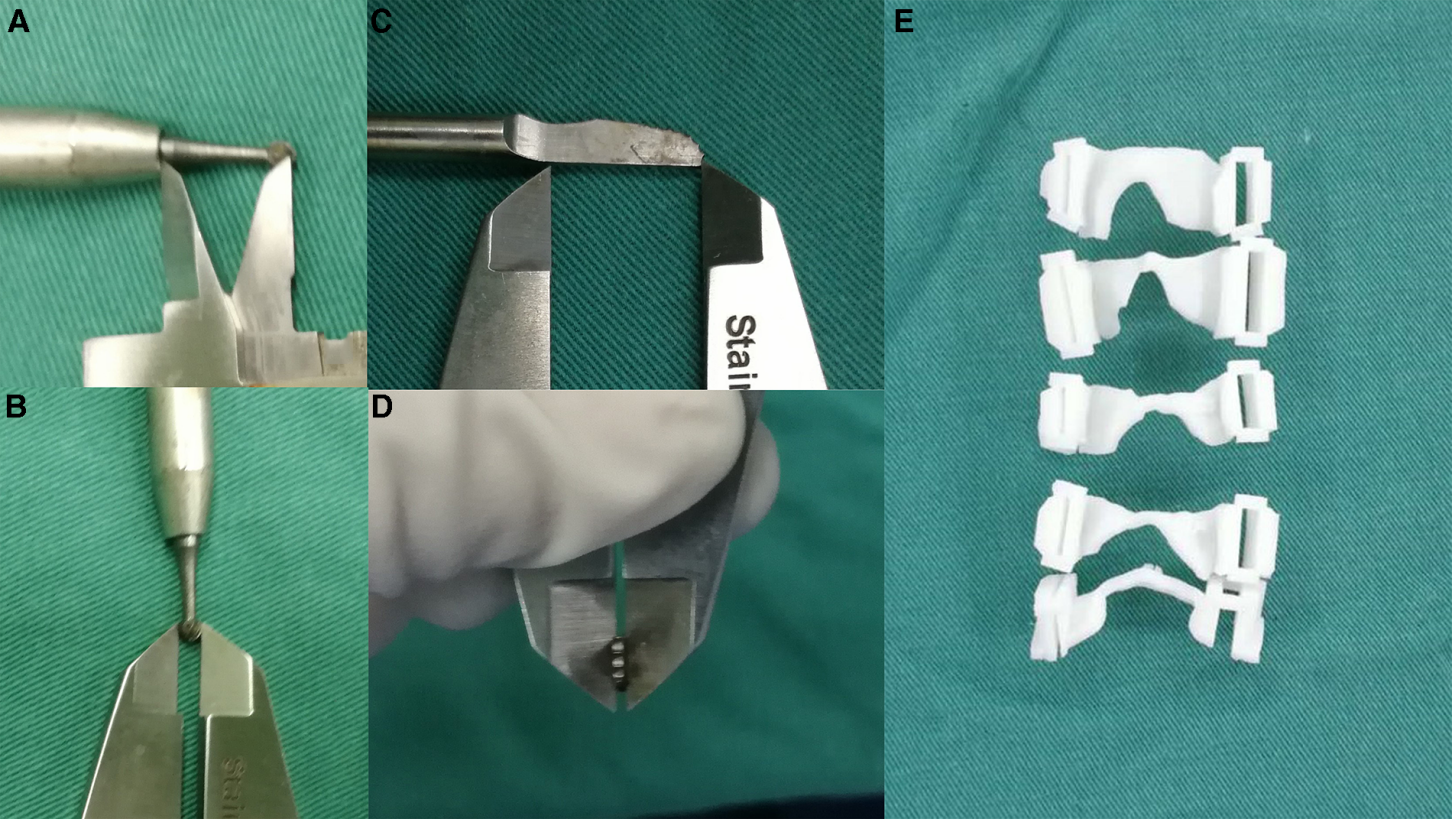
Template fabrication. (**A,B**) the measurement of the bur. (**C,D**) the measurement of the piezosurgery. (**E**) the 3D printed patient-specific drilling templates.

For each patient of the template group, the preoperative CT scan was imported into the commercially available software Mimics 17.0 (Materialize, Belgium) for the 3D reconstruction. Each vertebra from C3 to C7 was reconstructed and exported as “.stl” file. The “.stl” files were processed using the 3-Matic 9.0 software (Materialize, Belgium) for surgical simulation and template designing. Two cutting paths, slightly wider than the diameter of the bur used on the hinge side and the piezosurgery used on the open-door side, were simulated at the junction between the lamina and the lateral mass. The width and depth of the cutting path on the hinge side were based on the length and diameter of the bur (Figures 1A,B). The width and depth of the cutting path on the open-door side were based on the length and thickness of the piezosurgery (Figures 1C,D). These two cutting paths were also simulated to be 5 to 10 degrees deviated from the midline, according to the surgeon's preference. Next, part of the posterior surface of the lamina was extracted to create the body of the EOLP template, which was then subtracted by the two cutting paths. Thereafter, the EOLP template was exported as “.stl” file, which was processed by the 3D printer to fabricate the actual EOLP template in acrylate resin by stereolithography. Then, the templates were thoroughly washed and vacuum dried (Figure 1E). The 3D printed templates were sterilized with ethylene oxide and stocked in plastic bags.

### Surgical technique

**Figure 2:**
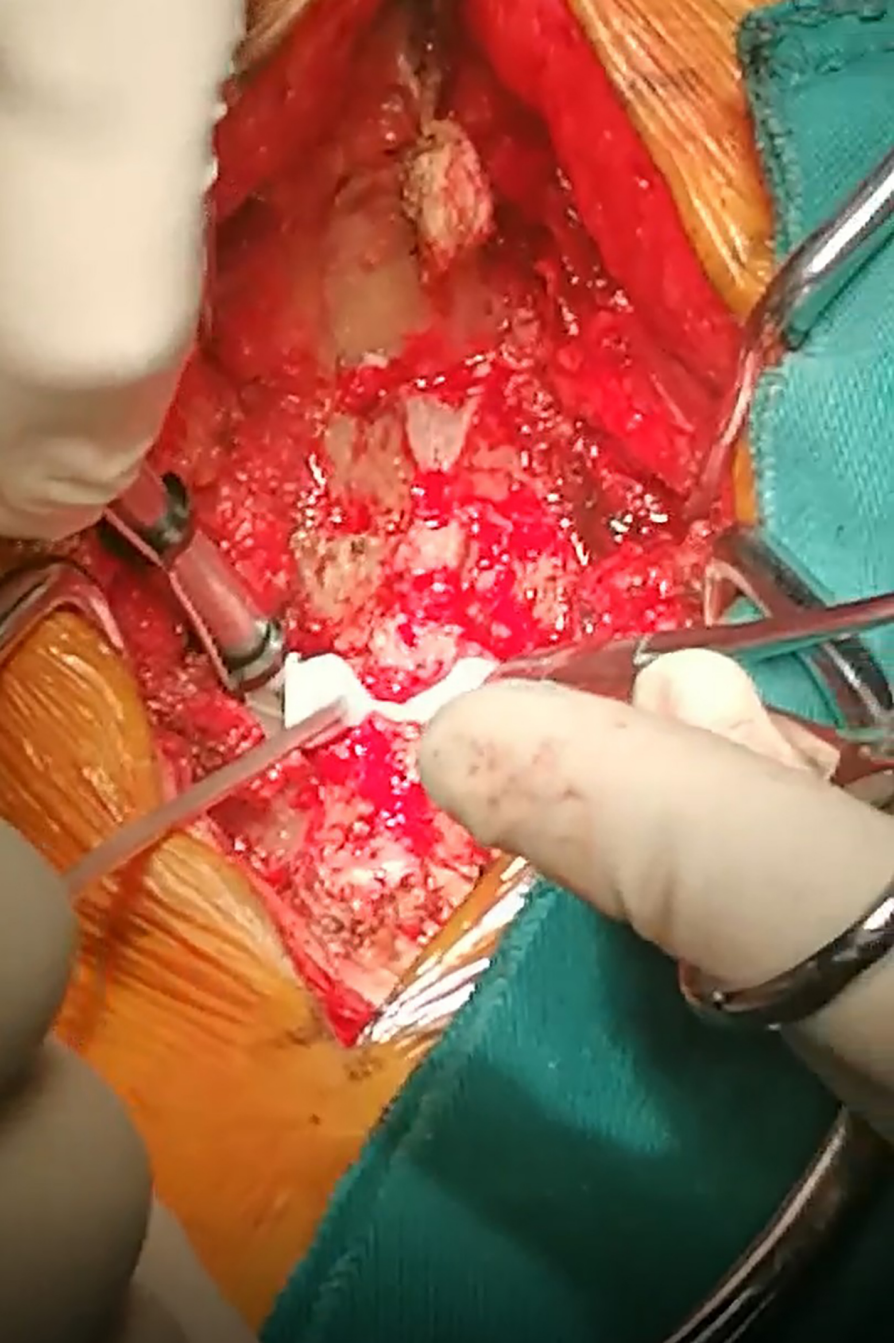
The application of the 3D printed patient-specific drilling templates during operation.

The procedure was carried out as described previously (10). Briefly, after general anesthesia, the patient was put in the prone position with the head fixed by the Mayfield holder. A midline incision was made to expose the spinous process and bilateral lamina from C3 to C7. The supraspinous ligament and interspinous ligament were then cut before the spinous processes were amputated. Thereafter, in the template group, the template was firmly pressed to the lamina. On the hinge side, the bur was placed through the path of the 3D-printed template and the trough was made by sliding the bur along the path. On the open side, the lamina was completely cut through the cutting path with piezosurgery (Figure 2). In the control group, the hinge side and open side were cut based on the bony landmarks and the surgeons' experience. Then the lamina was opened and carefully fixed with the Centerpiece mini-plate. The Centerpiece mini-plate was anchored to the lamina and lateral mass by two 5 mm screws and two 7 mm screws, respectively.

### Clinical evaluation

The neurological function was assessed by the Japanese Orthopedic Association (JOA) scores. Visual analogue scale (VAS) scores were used to evaluate neck pain intensity. The JOA and VAS scores were evaluated before surgery and at 12 months postoperatively. The occurrence of complications, including wound infection, cerebrospinal fluid (CSF) leakage, axial symptoms, and C5 palsy, were recorded and dealt with accordingly.

### Radiography study

**Figure 3:**
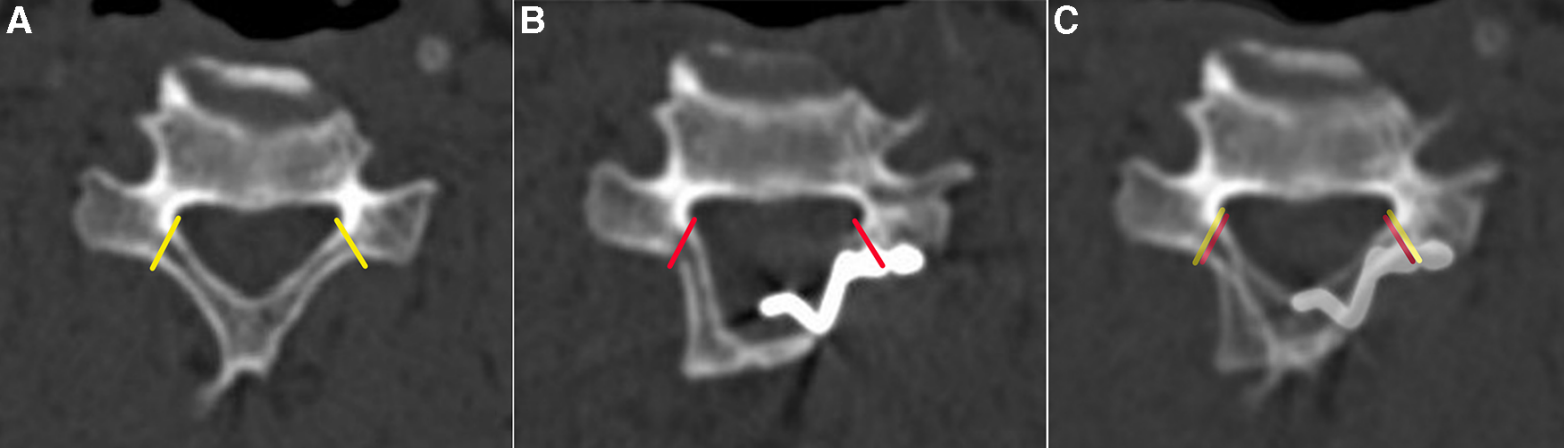
The method for the evaluation of the position of the hinge side and open-door side. (**A**) the satisfactory position was simulated at the transverse view of CT scans before surgery (the yellow lines). (**B**) the final position was marked at the transverse view of CT scans 3 days after surgery (the red lines). (**C**) **A** and **B** were overlapped to evaluate the deviation of the final position from the simulated position.

The C2–C7 Cobb angle and the C2–C7 range of motion (ROM) were measured using the Cobb method on the x-ray films before surgery and at 12 months postoperatively (11). The anteroposterior diameter of the canal and the Pavlov's ratio were determined on the lateral view of x-ray films before surgery, at 3 days postoperatively and 12 months postoperatively. The open angle was assessed using the axial view of CT scans taken 3 days postoperatively and 12 months postoperatively. The fracture type on the hinge side and the satisfaction of the trough position on the hinge side and open side were assessed using the axial view of CT scans taken 3 days postoperatively. It was deemed incomplete fracture if the ventral cortex was continuous. Otherwise, it was deemed complete fracture (6). The position of the hinge side and open-door side were evaluated in the axial view of CT scans before surgery and 3 days postoperatively. The satisfactory position was simulated at the axial view of CT scans before surgery in both groups. The final position was marked at the axial view of CT scans 3 days postoperatively. The deviation of the final position from the simulated position was recorded (Figure 3). If the deviation was less than 2 mm, it was recorded as satisfactory. Otherwise, it was recorded as unsatisfactory. The radiological evaluation was performed by two independent spine surgeons, and the mean values were used for statistical analysis.

### Statistical analysis

Statistical analysis was performed using SPSS software (version 19.0, SPSS Inc., IL, USA). The findings are presented as the mean values ± standard deviation (SD) or counts, as indicated. The paired Student's *t*-test was performed to test the difference before and after surgery within the groups. The independent Student's *t*-test and chi-square test were performed for analyzing the differences between the two groups. A value of *P* < 0.05 was considered statistically significant. A chi-square test or Fisher's exact test was used to analyze categorical data between the two groups.

## Results

### Patient information

The study started in August 2019 and ended in December 2020. A total of 40 patients were recruited. One patient in the template group and 2 patients in the control group were excluded because of the loss of follow-up. Other patients were all followed up for 12 months. The baseline demographic information for the two groups, including age, sex, body mass index (BMI), diagnosis, operative time, blood loss, size of mini-plate, preoperative VAS of neck and preoperative JOA were evaluated, and no significant differences were observed between the characteristics in the template group and the control group (*P* > 0.05). The occurrence rate of complications, including CSF leakage, axial symptoms and C5 palsy was a little higher in the control group, but no significant differences were observed (*P* > 0.05). All the complications were recovered within 1 month after surgery. No wound infection was observed in both groups. The details are described as follows (Table 1).

**Table 1:** Basic demographic information for the two groups.

	Template	Control	*P* value
Number of patients (*n*)	19	18	
Age (year)	55.74 ± 10.27	58.11 ± 10.74	0.497
Gender (Male/Female) (*n*)	12/7	10/8	0.638
BMI	24.34 ± 1.71	24.86 ± 2.02	0.402
Diagnosis (*n*)			0.821
MDH	10	8	
CCS	7	7	
OPLL	2	3	
Operative time (min)	137.10 ± 14.38	130.70 ± 10.79	0.141
Blood loss (ml)	230.50 ± 85.60	220.00 ± 97.20	0.728
Size of mini-plate			0.842
10 mm	8	7	
12 mm	11	11	
Complications (*n*)			0.687
CSF leakage	0	1	
Axial symptoms	1	2	
C5 palsy	1	1	
Preoperative VAS of neck	4.26 ± 1.59	4.50 ± 1.29	0.624
Preoperative JOA	8.26 ± 2.16	8.50 ± 2.09	0.737

BMI, body mass index; MDH, multi-level disc herniation; CCS, congenital cervical canal stenosis; OPLL, ossification of the posterior longitudinal ligament; CSF, cerebrospinal fluid; JOA, Japanese orthopedic association; VAS, visual analogue scale.

### Clinical results

In the template group, The VAS scores decreased significantly from 4.26 ± 1.59 preoperatively to 1.32 ± 0.86 at 12 months postoperatively (*P* < 0.05). The JOA scores increased significantly from 8.26 ± 2.16 preoperatively to 15.00 ± 1.73 at 12 months postoperatively (*P* < 0.05). In the control group, the VAS scores decreased significantly from 4.50 ± 1.29 to 2.06 ± 1.21, and the JOA scores increased significantly from 8.50 ± 2.09 to 14.61 ± 1.33 (*P* < 0.05). The significant difference of VAS scores between the two groups was observed at 12 months postoperatively (*P* < 0.05). The details are described as follows (Table 2).

**Table 2:** Comparison of the clinical outcomes between the two groups.

	Template	Control	*P* value
**VAS of neck**			
Preoperative	4.26 ± 1.59	4.50 ± 1.29	0.624
12 months postoperatively	1.32 ± 0.86*	2.06 ± 1.21*	**0** **.** **040** ^#^
**JOA**			
Preoperative	8.26 ± 2.16	8.50 ± 2.09	0.737
12 months postoperatively	15.00 ± 1.73*	14.61 ± 1.33*	0.451

* *P* < 0.05 compared to the preoperative parameter.

^#^ *P* < 0.05 between the template group and control group.

JOA, Japanese orthopedic association; VAS, visual analogue scale.

### Radiographic results

The C2–C7 Cobb angle and the C2–C7 ROM were decreased with no significant difference at 12 months postoperatively compared with that before surgery, and no significant difference was observed between the two groups (*P* > 0.05). The anteroposterior diameter and Pavlov's ratio increased significantly after surgery and remained well during the follow-up in both groups (*P* < 0.05). Besides, the data increased more in the template group compared with that in the control group with significantly differences at 3 days postoperatively and 12 months postoperatively (*P* < 0.05). The Open angle was also significantly higher in the template group compared with that in the control group at 3 days postoperatively and 12 months postoperatively (*P* < 0.05). The details are described as follows (Table 3).

**Table 3:** Comparison of the clinical outcomes between the two groups.

	Template	Control	*P* value
**C2–C7 Cobb (°)**			
Preoperative	15.97 ± 3.71	16.93 ± 3.29	0.409
12 months postoperatively	14.55 ± 3.09	14.83 ± 4.28	0.823
**C2–C7 ROM (°)**			
Preoperative	47.85 ± 14.53	49.98 ± 12.78	0.640
12 months postoperatively	38.77 ± 12.07*	41.87 ± 9.89*	0.401
**Anteroposterior diameter (mm)**			
Preoperative	10.57 ± 1.79	10.28 ± 2.01	0.639
3 days postoperatively	16.13 ± 1.70*	14.85 ± 1.65*	**0** **.** **026** ^#^
12 months postoperatively	16.10 ± 1.79*	14.71 ± 1.66*	**0**.**019**^#^
**Pavlov's ratio**			
Preoperative	0.54 ± 0.11	0.50 ± 0.10	0.259
3 days postoperatively	0.96 ± 0.10*	0.87 ± 0.10*	**0**.**011**^#^
12 months postoperatively	0.95 ± 0.10*	0.86 ± 0.09*	**0**.**007**^#^
**Open angle (°)**			
3 days postoperatively	32.92 ± 2.43	30.61 ± 2.75	**0**.**010**^#^
12 months postoperatively	33.32 ± 2.33	30.67 ± 2.90	**0**.**004**^#^

* *P* < 0.05 compared to the preoperative parameter.

^#^ *P* < 0.05 between the template group and control group.

ROM, range of motion.

A total of 185 lamina were analyzed. Based on the CT scans taken 3 days after surgery, 37 out of 90 (41.11%) were completely fractured on the hinge side in the control group, which was significantly higher compared with that in the template group (*P* < 0.05) (Table 4). The satisfaction of the trough position on both sides was 87.37% (166/190) in the template group, which was significantly higher than that in the control group (*P* < 0.05) (Table 5).

**Table 4:** Comparison of the fracture type between the two groups.

	Template	Control	*χ* ^2^	*P* value
Incomplete fracture	78 (82.11%)	53 (58.89%)	12.052	**0.0005**
Complete fracture	17 (17.89%)	37 (41.11%)		

**Table 5:** Comparison of the satisfaction of the trough position on both sides between the two groups.

	Template	Control	*χ* ^2^	*P* value
Satisfactory	166 (87.37%)	130 (72.22%)	13.253	**0.0003**
Dissatisfactory	24 (12.63%)	50 (27.78%)		

### Analysis in senior and junior surgeons

Nine patients in the template group and 9 patients in the control group were performed by the senior surgeon, and 10 patients in the template group and 9 patients in the control group were performed by the junior surgeon. The incomplete fracture rate and satisfaction of the trough position on both sides had no significant difference between the two groups when the EOLP was performed by the senior surgeon (*P* > 0.05). However, to the junior surgeon, the incomplete fracture rate and satisfaction were significantly higher in the template group compared with that in the control group (*P* < 0.05). The details are described as follows (Tables 6, 7).

**Table 6:** Comparison of the fracture type between the two surgeons.

	Senior surgeon				Junior surgeon			
	Template	Control	*χ* ^2^	*P* value	Template	Control	*χ* ^2^	*P* value
Incomplete fracture	37 (82.22%)	31 (68.89%)	2.166	0.1411	41 (82.00%)	22 (48.89%)	11.624	**0.0007**
Complete fracture	8 (17.78%)	14 (31.33%)			9 (18.00%)	23 (51.11%)		

**Table 7:** Comparison of the satisfaction of the trough position on both sides between the two surgeons.

	Senior surgeon				Junior surgeon			
	Template	Control	*χ* ^2^	*P* value	Template	Contro	*χ* ^2^	*P* value
Satisfactory	80 (88.89%)	72 (80.00%)	2.707	0.0999	86 (86.00%)	58 (64.44%)	11.995	**0.0005**
Dissatisfactory	10 (11.11%)	18 (20.00%)			14 (14.00%)	32 (35.56%)		

## Discussion

The making of the open-door side cutting path and hinge side trough was the most technically demanding procedure during the EOLP surgery. The proper position and depth of both sides were reported to be related to the clinical outcomes and complications (12, 13). Ideally, the open-door side cutting path should be made at the junction between the lamina and the lateral mass with the cutting off of the ventral cortex appropriately. The hinge side trough should be made at the junction between the lamina and the lateral mass with the dorsal cortex and cancellous bone removed, keeping the ventral cortex intact. However, achieving such a target is technically demanding. Accurately locating the position and appropriately cutting the bone could decrease the incidence of hinge side complete fracture, achieve sufficient decompression and ensure the safety of the surgery (14–16).

The complete fracture of the lamina on the hinge side compromised the stability of the lamina (17). Under the push and pull effect of the posterior cervical soft tissue, the floppy lamina could be displaced. The displacement of the lamina into the spinal canal could compress the spinal cord or encroach the nerve root, causing deterioration of the neurological functions. We previously reported the incidence of complete fracture to be 56.8%, among which 3.68% of the lamina displaced into the canal (6). Although no spinal disorders were detected in these patients during the follow-up, longer observation was still needed.

The 3D printing technique has been recently applied in surgery (18, 19). This technique has been tested both *in vitro* and clinically for posterior cervical surgery, especially for the pedicle screw placement (20, 21). In these cases, the entry point and trajectory of the screws were planned before surgery, and the templates were modeled in the computer software. The templates can be designed for a specific patient according to his or her anatomical features (7). Thus, it is especially useful for the surgeries performed on the site of anatomical abnormality or variation. In our study, the 3D printed patient-specific drilling template could help the surgeon locate the trough position and control the depth more properly.

With the aid of the 3D printed templates with the function of locating and depth control, the patients could achieve a better relief of the neck pain. That may attribute to the accurate slotting on the hinge side which achieved a high fusion rate at 12 months postoperatively. Besides, the anteroposterior diameter, Pavlov's ratio and Open angle were all increased in the template group, which meant the decompression was more expansive with the aid of the templates. The satisfaction of the trough position on the open-door side and hinge side and the incomplete fracture rate on the hinger side were higher in the template group, which meant the templates used in the template group could improve the accuracy of locating and depth control. This phenomenon may not be outstanding to the senior surgeon because of his experienced technique. But to the junior surgeon, the effect was remarkable.

There is a limitation in our study. This is a single-center, prospective randomized controlled study with a relatively small sample size. In the future, a multi-center prospective randomized controlled study with a larger sample size should be performed to further validate the results of our study.

## Conclusion

The application of 3D printed patient-specific drilling templates with the function of locating and depth control for EOLP could improve the outcome of neck pain relief and expand the decompression. It can also improve the satisfaction of the trough position on the open-door side and the hinge side and decrease the complete fracture rate on the hinge side, especially for the junior surgeon.

## Data Availability

The raw data supporting the conclusions of this article will be made available by the authors, without undue reservation.
